# Dietary fibre and the gut–brain axis: microbiota-dependent and independent mechanisms of action

**DOI:** 10.1017/gmb.2021.3

**Published:** 2021-09-08

**Authors:** Danique La Torre, Kristin Verbeke, Boushra Dalile

**Affiliations:** Translational Research Center in Gastrointestinal Disorders (TARGID), Department of Chronic Diseases and Metabolism, Faculty of Medicine, KU Leuven, Leuven, Belgium; Leuven Brain Institute, KU Leuven, Leuven, Belgium

**Keywords:** Dietary fibres, prebiotics, SCFA, affective processes, cognition

## Abstract

Dietary fibre is an umbrella term comprising various types of carbohydrate polymers that cannot be digested nor absorbed by the human small intestine. Consumption of dietary fibre is linked to beneficial effects on cognitive and affective processes, although not all fibres produce the same effects. Fibres that increase short-chain fatty acid (SCFA) production following modulation of the gut microbiota are thought to be the most potent fibres to induce effects on cognitive and affective processes. SCFAs can exert their effects by improving central, peripheral and systemic immunity, lowering hypertension and enhancing intestinal barrier integrity. Here, we propose additional mechanisms by which dietary fibres may contribute to improvements in affective and cognitive processes. Fibre-induced modulation of the gut microbiota may influence affective processes and cognition by increasing brain-derived neurotrophic factor levels. Depending on the physicochemical properties of dietary fibre, additional effects on affect and cognition may occur via non-microbiota-related routes, such as enhancement of the immune system and lowering cholesterol levels and subsequently lowering blood pressure. Mechanistic randomised placebo-controlled trials are needed to establish the effects of dietary fibre consumption and the magnitude of explained variance in affect and cognition when incorporating measurements of microbiota-dependent and microbiota-independent mechanisms in humans.

## Introduction

Dietary fibre, which is derived from fruits and vegetables, legumes, whole-grain breads, and cereals, is an umbrella term comprising various types of carbohydrate polymers that cannot be digested nor absorbed by the human small intestine. Consequently, dietary fibre ends up in the colon, where it is fermented by the gut microbiota. Consumption of dietary fibre has been linked to various beneficial physiological effects, including effects on affective (Lawton et al., 2013) and cognitive (Sandberg et al., 2018) processes. Notably, dietary fibre consumption can aid in prevention or treatment of symptoms of depression, anxiety, and stress (Jacka et al., 2017; Silk et al., 2009), which are psychological states characterised by negative mood (e.g., worry, fear, sadness, loss of interest, or emotional tension) that are often accompanied by impaired facets of cognition. Moreover, preclinical data suggests that impaired facets of cognition, such as attention, mental flexibility, and executive functioning, can be restored by dietary fibre (Chunchai et al., 2018).

Since dietary fibres vary in origin, chemical composition, and physicochemical properties (comprising solubility, fermentability, and viscosity), they can exert different effects on the body. These differences in physicochemical properties might differentially affect the communicative pathways to the brain, and consequently variably influence affective and cognitive processes. Commonly accepted mechanisms by which dietary fibres are thought to affect cognitive and affective processes are the gut microbiome and short chain fatty acids (SCFAs) (Dalile et al., 2019). Here, we shed light on additional potential mechanisms that may contribute to the positive effects of dietary fibres on these processes, such as the immune system, cholesterol, the intestinal barrier, brain-derived neurotrophic factor (BDNF), and blood pressure, and would help explain additional variance that is not explained by SCFAs. Since in human studies most evidence is associative, we also included animal studies that assess the impact of dietary fibre intake on these various mechanisms. To this end, the current review assesses microbiota-dependent and microbiota-independent biological mechanisms that may underlie the effects of dietary fibre on affective processes and cognition and outlines which physicochemical properties may predict such effects.

## Dietary fibres: a definition

Since dietary fibres are characteristically heterogeneous, different classifications have been used to define them, including origin, chemical composition, and physicochemical properties with additional subcategorisation based on the degree of polymerisation. The most commonly used definition of dietary fibre is according to the Codex Alimentarius, which states that dietary fibres include (1) edible carbohydrate polymers occurring in foods as consumed, (2) edible carbohydrates obtained from food raw materials by physical, enzymatic or chemical means and (3) synthetic carbohydrate polymers, which have beneficial physiological effect demonstrated by generally accepted scientific evidence (Joint FAO/WHO Food Standards Programme Secretariat of the CODEX Alimentarius Commission, 2010). In 2016, the Food and Drug Administration (FDA) defined dietary fibre as either (1) non-digestible soluble and insoluble carbohydrates (with three or more monomeric units), and lignin that are intrinsic and intact in plants, or (2) isolated or synthetic non-digestible carbohydrates (with three or more monomeric units), and induce physiological effects that are beneficial to human health (Food and Drug Administration, 2020). At that time, only seven non-digestible carbohydrates were included in the latter category (β-glucan soluble fibre, psyllium husk, cellulose, guar gum, pectin, locust bean gum, and hydroxypropylmethylcellulose). Since then, the list has been extended with ten additional types of dietary fibre [mixed plant cell wall fibres, arabinoxylan (AX), alginate, inulin, and inulin-type fructans (ITFs), high amylose starch (resistant starch 2), galactooligosaccharides (GOS), polydextrose, resistant maltodextrin/dextrin, cross linked phosphorylated RS4, and glucomannan]. Certain types of fibres, such as ITFs and GOS fulfil the criteria of a prebiotic. Prebiotics are defined as “a substrate that is selectively utilised by host microorganisms conferring a health benefit” (Gibson et al., 2017).

### Properties of dietary fibres

The physicochemical characteristics of dietary fibres are subtyped by solubility, viscosity and fermentability. Table 1 provides an overview of the most frequently investigated types of fibres and their concomitant physicochemical properties.

**Table 1.**
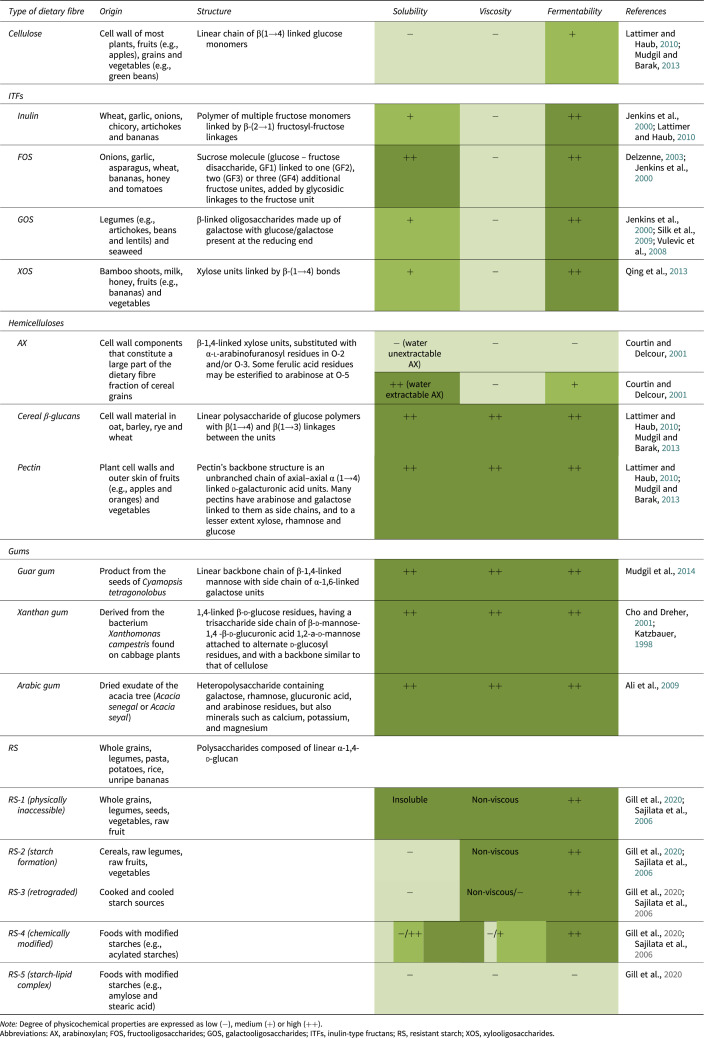
Overview of different types of dietary fibres with their concomitant origin, structure and degree of solubility, viscosity and fermentability.

#### Solubility

Dietary fibres can be water-soluble or water-insoluble, with most plant foods containing a mixture of both. Approximately 20 per cent of consumed dietary fibre is soluble, whereas 80 per cent is insoluble. Insoluble fibres consist mainly of cellulose, hemicellulose, and lignin, and are primarily present in wheat bran, most grain products and vegetables (Cho and Dreher, 2001). Soluble fibres consist of polysaccharides such as pectin, mucilage, and gum, and are predominantly found in some fruits (e.g., dried prunes, oranges, and grapefruit), oats, barley, dried beans, and legumes (e.g., lentils and pinto beans) (Cho and Dreher, 2001). Although soluble fibres are less prevalent in foods than insoluble fibres, they have an important influence on digestive and absorptive processes, such as delaying gastric emptying, decreasing glucose absorption, enhancing immune function, and lowering total and low-density lipoprotein (LDL) cholesterol levels (Cho and Dreher, 2001; Jenkins et al., 2000). Insoluble fibres are important for shortening bowel transit time, increasing faecal bulk, and softening stool (Cho and Dreher, 2001).

#### Viscosity

Viscosity, the capacity to gel with water, depends on the solubility of the fibre with soluble fibres having higher viscosity. Viscous fibres reduce postprandial glucose response after carbohydrate-rich meals, lower total and LDL cholesterol levels (Jenkins et al., 2000; McRorie and McKeown, 2017), and slow gastric emptying and macronutrient absorption from the gut (McRorie and McKeown, 2017).

#### Fermentability

The fermentability of dietary fibre varies greatly, ranging from not at all (e.g., lignin) to almost complete fermentation (e.g., pectin) (Mudgil and Barak, 2013). Soluble fibres are fermented in the colon, whereas insoluble fibres are less and more slowly fermented. The fermentation of soluble fibres results in the production of short-chain fatty acids (SCFAs) which, in turn, exhibit anti-inflammatory effects in the gut, maintain intestinal barrier integrity (Lewis et al., 2010), induce mucin secretion in the gastrointestinal tract (Monk et al., 2017), and promote gastrointestinal motility (Cherbut et al., 1998). In addition to solubility, chain length and particle size determine fermentability with shorter polymers and small particles being more readily fermented. In *in vitro* incubation experiments, neutral pectin fractions produced more SCFAs and more acetate than acidic fractions, whereas rhamnose and arabinan produced high proportions of propionate (Onumpai et al., 2011). Wood derived xylan and mannan derivatives produced similar total amounts of SCFAs as fructooligosaccharides (FOS) but proportionally more propionate and butyrate, respectively (La Rosa et al., 2019). In contrast, the glycosidic bond configuration has little impact on SCFA production (Harris et al., 2017).

## Dietary fibre studies on cognitive and affective processes

Here, we provide a brief overview of the state of the literature on the effects of dietary fibre on cognitive and affective processes for the sake of completeness. Cross-sectional and prospective cohort studies across the lifespan continue to show positive associations between higher dietary fibre intake and better cognitive and affective processes (Muth and Park, 2021; Swann et al., 2020). Affective processes are measured by, for instance, better mental health quality of life and lower incidents of depression and anxiety. Moreover, dietary source of fibres appears to be differentially associated with the incidence of depression (Kim et al., 2020). Despite assessing effects on the short-term, human interventional studies enable controlled administration of specific fibres and exclusion of confounding effects. These studies are displayed in Table 2. Briefly, 18 studies were found, with 7 of them (Azpiroz et al., 2017; Buigues et al., 2016; Dehhaghi et al., 2019; Farhangi et al., 2018; Grimaldi et al., 2018; Kao et al., 2019; Silk et al., 2009) conducted in populations with various mental, physical, or gastrointestinal disorders, and the remaining 11 conducted in healthy populations (Best et al., 2008, 2009, 2015; Childs et al., 2014; Lawton et al., 2013; Pasman et al., 2003; Ramnani et al., 2015; Schmidt et al., 2015; Smith, 2005; Smith et al., 2015; Talbott and Talbott, 2009). The duration of the intervention periods varied, with three studies carrying out single administration (Best et al., 2008, 2015; Smith et al., 2015), one study comprising 2 days (Pasman et al., 2003), and the others comprising 2–13 weeks (Azpiroz et al., 2017; Best et al., 2009; Buigues et al., 2016; Childs et al., 2014; Farhangi et al., 2018; Grimaldi et al., 2018; Kao et al., 2019; Kazemi et al., 2019; Lawton et al., 2013; Ramnani et al., 2015; Schmidt et al., 2015; Silk et al., 2009; Smith, 2005; Talbott and Talbott, 2009). Eleven studies utilised ITFs, with the majority of them (nine studies) showing some positive effects on affective and cognitive indices. Five studies utilised mixtures of fibre, mostly revealing positive effects of interventions, unless it was administered only once. One study administrated β-glucans and one study administered resistant starch. Not all studies are of equal quality, with some lacking proper control conditions. Notably, most human intervention studies administered dietary fibre supplements rather than whole foods. Whole foods are not only rich in dietary fibre but also contain minerals and antioxidants that may improve mood and cognition (Gomez-Pinilla, 2008). Moreover, it cannot be excluded that some placebos (e.g., maltodextrin) may have had an effect on mood and/or cognition as well, possibly via gut-independent mechanisms (Kendig et al., 2014). Future intervention studies should take such effects into account. Some fibres such as pectin, hemicelluloses (e.g., AX), and gums (e.g., guar, xanthan, and Arabic gum) are yet to be explored in the context of mood and cognition. As these fibres are highly viscous, they may affect mood and cognition through microbiota-independent mechanisms as discussed below. Overall, the majority of studies point to beneficial effects on mood and cognition, but none of these studies causally addresses potential mechanisms of action. In the next section, we will discuss potential mechanisms, both microbiota-dependent as well as microbiota-independent, that may be involved in dietary fibre’s beneficial effects on affective and cognitive processes.

**Table 2.** Human studies assessing the effect of dietary fibre interventions on mood and cognition.

References	Sample	Design	Intervention	Placebo	Effect
Pasman et al. (2003)	26 healthy men (25–45 years)	A 2-day, open, randomised, crossover trial	Simple carbohydrate breakfast (high in mono and disaccharides; 2.1 g fibre) versus complex carbohydrate breakfast (high in polysaccharides; 6.5 g fibre)	None	Complex carbohydrate breakfast lowered fatigue scores relative to simple carbohydrate breakfast. No effects on depression, anger, vigour or tension scores
Smith (2005)	142 healthy participants (19–64 years)	A 2-week, placebo-controlled, crossover design with 2 weeks washout period	2 × 5 g/day oligofructose-enriched inulin	Placebo (no further details)	Oligofructose-enriched inulin decreased simple and choice reaction time relative to placebo. No other effects
Best et al. (2008)	45 healthy participants (40–63 years)	An acute randomised, double-blind, placebo-controlled design	7 g powdered Ambrotose® Complex (combination of saccarides). These include polysaccharides from aloe vera, *Larix decidua, Astragalus gummifer* and *Anogeissus latifolia*, with rice starch and glucosamine hydrochloride; and saccharides included mannose, galactose, fucose, xylose, glucose, *n*-acetyl-glucosamine, *n*-acetyl-neuraminic acid and *n*-acetyl-galactosamine	25 g of glucose or artificial sweetener (two drops of liquid Stevia)	No effects
Best et al. (2009)	109 healthy participants (45–60)	A 12-week randomised, double-blind, placebo-controlled	3.6 g/day powdered Ambrotose® complex (a combination of plant saccharides). These include polysaccharides from aloe vera, *Larix decidua, Astragalus gummifer* and *Anogeissus latifolia*, with rice starch and glucosamine hydrochloride; and saccharides included mannose, galactose, fucose, xylose, glucose, *n*-acetyl-glucosamine, *n*-acetyl-neuraminic acid, and *n*-acetyl-galactosamine	Rice flour starch powder	Ambrotose complex improved memory performance (immediate recall and recognition memory), well-being (reduction in anger-hostility and depression–dejection, feeling less irritable, having fewer instance of being grouchy and annoyed, and overall more positive and happier, experience fewer feelings of personal inadequacy) relative to placebo
Talbott and Talbott (2009)	75 marathon runners (18–53)	A 4-week randomised, double-blind, placebo-controlled trial	250 or 500 mg/day of β 1,3/1,6 glucan. The β-glucan were isolated from the yeast *Saccharomyces cerevisiae*	Rice flour	Lower tension, fatigue, confusion, and anger and higher vigour scores in the treatment groups relative to placebo – with some exhibiting dose-depending effects. A composite (global mood) score revealed that mood improved after both the 250 mg dose at 4-weeks and both 2- and 4-weeks in the 500 mg treatment groups compared to placebo
Silk et al. (2009)	Patients with irritable bowel syndrome (18–80 years)	A 12-week randomised, simple blind (patients), placebo-controlled, parallel, and crossover design	Trans-GOS mixture (3,5 or 7 g/d), which contained: GOS, lactose, glucose, and galactose	Maltodextrin	Trans-GOS mixture decreased anxiety and increased quality of life scores relative to placebo
Lawton et al. (2013)	153 healthy low-fibre (<15 g/day) consumers (18–50 years)	A single centre, multi-site, open, within-subjects pre-post design. A 14-day non-intervention (baseline, habitual diet) monitoring period was followed by a 14-day fibre consumption (intervention) period	Cereals (5.4 g of fibres with 3.5 g of wheat bran). The source of fibres was from bran shreds, wheat bran flakes, wheat bran flakes with sultanas, frosted mini wheats, raisin mini wheats, chocolate wheat bran flakes, apple and fig wheat bran flakes	No comparator (non-intervention, baseline monitoring period)	Breakfast cereals increased subjective perception of general well-being (feeling less fat, more mentally alert, slim, happy, and energetic whilst experiencing less stress, mental and physical tiredness, difficulty concentrating, and fewer headaches)
Childs et al. (2014)	44 healthy participants (25–65 years)	A 3-week randomised, double-blind, placebo-controlled, crossover design with 2-week washout period	Prebiotic: 8 g/day XOS; Probiotic: *Bifidobacterium animalis* subsp. *lactis* (Bi-07; 109 colony-forming units (CFU)/d); or, Synbiotic: combined	Maltodextrin	XOS increased participant-reported vitality and happiness relative to placebo
Smith et al. (2015)	47 healthy participants (19–30)	An acute randomised, double-blind, placebo-controlled crossover design	5 g/day Inulin	Maltodextrin	Inulin resulted in higher happiness scores, and improved memory [greater accuracy on a recognition memory task, albeit with slower reaction time, and improved recall performance (immediate and delayed)] relative to placebo
Schmidt et al (2015)	45 healthy volunteers (18–45 years)	3-week, double-blind, randomised, placebo-controlled trial	5.5 g/day FOS or Bimuno®-GOS (B-GOS®)	Maltodextrin	B-GOS® lowered attentional vigilance to negative versus positive stimuli on dot-probe task as well as lowered cortisol awakening response (CAR)^a^ relative to placebo. No effects of FOS
Ramnani et al. (2015)	38 healthy participants (18–50 years, mean age 38)	A 3-week randomised, double-blind, placebo-controlled, crossover design with 2-week washout period	Agave fructan (5 g/day; a purified powder extracted from *Agave tequilana* Weber var. A*zul.*)	Maltodextrin	No effects
Best et al. (2015)	73 healthy participants (45–60 years)	An acute randomised, double-blind, placebo-controlled trial	4 g of a proprietary mixture of non-starch polysaccharides (NSPs; Ambrotose® complex). This included arabinogalactan, aloe vera gel extract, gum ghatti, gum tragacanth that contain gluco- and galactomannans, acetylated mannans, acemannan, glucosamine HCL, and rice starch	Rice flour or sucrose control	Non-starch polysaccharides resulted in glucose-independent improvements in recognition memory and working memory relative to rice flour and sucrose control
Buigues et al. (2016)	Participants with frailty syndrome (66–90 years)	A 13-week randomised, double-blind, placebo-controlled trial	7.5 g/day Inulin plus FOS (Darmocare Pre)	Maltodextrin	Inulin + FOS reduced fatigue relative to placebo
Azpiroz et al. (2017)	79 irritable bowel syndrome patients with rectal hypersensitivity (18–60 years old)	4-week, double-blind, randomised, placebo-controlled trial	5 g/day short chain FOS (scFOS)	Maltodextrin	scFOS lowered anxiety scores relative to placebo
Farhangi et al. (2018)	55 females with type 2 diabetes mellitus	8-week, double-blind, randomised, placebo-controlled trial	10 g/day Nutriose®06 (a resistant dextrin), which contains glucose polymer derived from maize, wheat, or other edible starch	Maltodextrin	Resistant dextrin lowered cortisol levels, depression, anxiety, and stress scale scores, and improved scores on general health questionnaire relative to placebo
Grimaldi et al. (2018)	26 children (4–11 years) with autism spectrum disorders	6-week randomised, double-blind, placebo-controlled trial	B-GOS® mixture (Bimuno; 1.8 g: 80% GOS content)	Maltodextrin	B-GOS® improved social behaviour scores (i.e., scores were lower) relative to placebo
Kazemi et al. (2019)	110 participants with major depressive disorder (18–50 years)	8-week randomised, double-blind, placebo-controlled trial	*L. helveticus* R0052 and *B. longum* R0175 (CNCM strain I-3470) bacteria at a dosage of 10 billion colony-forming units (≥10 × 10^9^ CFU) per 5 g sachet/day	Excipients used were as follows: xylitol, maltodextrin, plum flavour and malic acid	Probiotic supplementation resulted in a significant decrease in depression score compared to the placebo and prebiotic supplementation
			Prebiotic product was composed of GOS and 0.2% Plum flavour		
Kao et al. (2019)	39 non-hospitalised participants (18–60 years) with psychosis, stable on antipsychotic medication for at least 3 months prior to recruitment, with global cognitive score 0.5 SD below the healthy average	A 12-week randomised, double-blind, placebo-controlled crossover trial, without washout period	5.5 g/day B-GOS®	Maltodextrin	B-GOS® increased the cognitive assessment’s composite score (reflecting verbal memory, working memory, motor speed, verbal fluency, attention, speed of information processing, and executive functions) relative to placebo. Placebo improved verbal fluency alone. There was a significant pre- versus post-effect of B-GOS® on the executive, but not verbal, domains

Abbreviations: GOS, galactooligosaccharides; FOS, fructooligosaccharides; XOS, xylooligosaccharides.

a CAR a measure of hypothalamic–pituitary–adrenal (HPA) axis function (Clow et al., 2010). CAR has been previously associated with anticipation of daily stress (Fries et al., 2009) and executive function (Butler et al., 2017).

## Biological mechanisms

**Figure 1:**
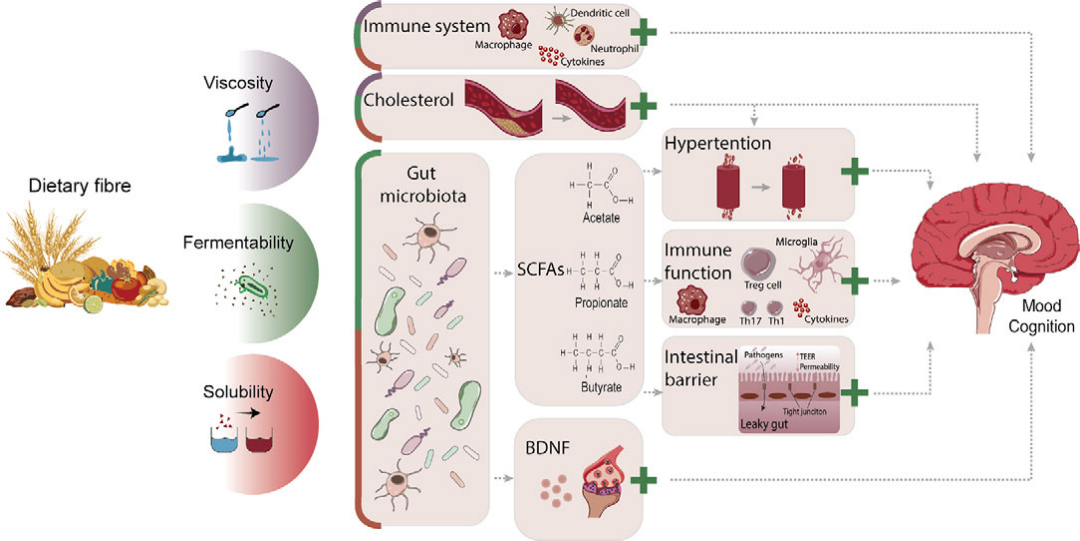
Overview of potential biological mechanisms underlying dietary fibres’ effects on mood and cognition according to physicochemical properties. The degree of viscosity, fermentability, and solubility of dietary fibres influences gut microbiota composition and function, the immune system, as well as cholesterol levels, through which mood and cognition can be modulated. Via the direct immune route, viscous, fermentable, and soluble fibres can reduce pro-inflammatory cytokines, lower numbers and activation of splenic macrophages and DCs, and increase neutrophils in the blood. Cholesterol levels can be lowered by viscous, fermentable, and soluble fibres and impact the brain, but can also indirectly affect it by reducing hypertension. Fermentability and solubility of dietary fibre modulate the gut microbiota, by which it can influence affective and cognitive processes via SCFA-dependent mechanisms or BDNF levels. SCFAs can lower hypertension as well as improve immune function and intestinal barrier integrity. Via the indirect immune route, SCFAs suppress pro-inflammatory reactions by reducing pro-inflammatory cytokines, increasing anti-inflammatory cytokines and T_reg_ cells, as well as restoring microglial cell morphology and reversing microglial immaturity. All these mechanisms have been associated with modulation of mood and cognition in health and disease. The likelihood that the different biological mechanisms underlie dietary fibres’ effect on mood and cognition are displayed in proportion, with larger cubicle areas reflecting increased potential. The green plus sign indicates beneficial effects. BDNF, brain-derived neurotrophic factor; DCs, dendritic cells; T_reg_ cells, regulatory T-cells; SCFAs, short chain fatty acids.

Dietary fibre may affect mood and cognition via various biological mechanisms, both microbiota-dependent and microbiota-independent, which are reviewed in this section. Figure 1 provides an overview of the different biological mechanisms that may underlie dietary fibres’ effect on affective and cognitive processes according to their physicochemical properties.

### Gut microbiome

Dietary fibre’s ability to influence affective and cognitive processes is most likely through its interactions with the gut microbiome. The gut microbiome refers to the 1 × 10^13^ to 1 × 10^14^ microorganisms that reside in the human large intestine, which is one of the most densely and diversely colonised organs in the human body. Pre-clinical studies suggest that the gut microbiome plays an important modulating role in affective and cognitive processes. Studies in germ-free (GF) mice suggested a causal role of the gut microbiota in developing brain function and behaviour, as those mice exhibit compromised social, cognitive, and anxiety-like behaviours (Desbonnet et al., 2014), such as reduced non-spatial memory, lower social motivation, and symptoms of anxiety compared to their conventionally raised specific pathogen-free (SPF) counterparts (Clarke et al., 2013; Desbonnet et al., 2014). Also, faecal microbiota transplantation of “depressive microbiota” derived from patients with major depressive disorder (MDD; a disorder specifically known for its symptoms of negative mood and impaired cognition) to GF mice (Zheng et al., 2016) resulted in depression-like behaviours relative to colonisation with “healthy microbiota” derived from healthy donors.

Human studies also suggest that differences in the gut microbiome composition are associated with mood and cognition. For instance, gut microbiota composition of healthy subjects differs from that of patients suffering from MDD. Specifically, the relative abundance of *Firmicutes* (Jiang et al., 2015), *Bifidobacteria* and *Lactobacilli* (Aizawa et al., 2016) appears lower in MDD patients compared to healthy controls, whereas *Actinobacteria*, *Proteobacteria*, and *Bacteroidetes* appear to be higher (Jiang et al., 2015). Increases in the relative abundance of *Bifidobacteria* and *Lactobacilli* in particular have been associated with lower anxiety and better memory and learning in both rodents (Bravo et al., 2011) and humans (Messaoudi et al., 2011). Nevertheless, it seems unlikely that bacterial strains are directly responsible for these effects. Rather, it is speculated that bacterial metabolites like SCFAs and BDNF mediate the interaction between the microbiota and psychological functioning.

#### Short-chain fatty acids

Soluble fibres (e.g., pectin and gums) provide substrates for bacterial fermentation, which in turn leads to the production of SCFAs in the colon. Acetate, propionate, and butyrate are the most abundant SCFAs, and they are present in the colon in an approximate molar ratio of 60:20:20, respectively. Type and availability of substrate, as well as gut transit time and composition of the gut microbiota influence the levels and relative proportions of SCFAs in the colon. Following their production by microbial fermentation of dietary fibre, SCFAs are rapidly absorbed by colonic cells via monocarboxylate transporters and produce energy for the cells. Those that are not absorbed travel via the basolateral membrane into the portal circulation. In the liver, SCFAs are incorporated in glucose, cholesterol, and fatty acids, thus only small amounts actually reach systemic circulation, and subsequently the brain (Boets et al., 2017).

##### SCFAs and cognitive and affective processes

While numerous animal studies have relied on intracerebroventricular (ICV), intraperitoneal, subcutaneous, and oral administrations to deliver SCFAs and investigate their impact on brain and behaviour (see Dalile et al., 2019), few studies explored whether the effects of dietary fibre/prebiotics on affective and cognitive functioning are mediated by or correlated with the observed increases in SCFA levels. One of these studies showed that FOS- and GOS-induced increases in cecal SCFAs correlated with effects on depressive and anxious behaviour and stress responses in mice (Burokas et al., 2017). Moreover, drinking water supplemented with a GOS mixture (B-GOS; 3 per cent) for 3 weeks increased plasma acetate levels and upregulated subunits of the *N*-methyl-d-aspartate receptor (NMDAR) in the brain (Gronier et al., 2018; Kao et al., 2018), which is implicated in synaptic plasticity and memory formation, as well as Acetyl Co-A Carboxylase mRNA, an enzyme in the brain that plays an important role in supplying fatty acids for myelination. Indeed, in one of these studies, mice fed with the GOS mixture gained greater cognitive flexibility (Gronier et al., 2018). Unfortunately, the other did not test whether the GOS mixture induced changes in cognition (Kao et al., 2018). Direct administration of acetate also induced similar neurochemical changes as that of GOS (Gronier et al., 2018), suggesting that acetate may play a mechanistic role in the observed effects of GOS administration.

Unfortunately, studies in humans administering prebiotics and assessing effects on affective and cognitive processes do not quantify SCFAs and do not explore the extent to which circulating SCFAs mediate the observed effects (Dalile et al., 2019). However, one study found that increasing colonic propionate by consumption of 10 g of an inulin–propionate ester (thereby delivering of 2.36 g propionate to the colon, which is 2.5 times habitual daily propionate production) influenced brain anticipatory reward responses during a functional magnetic resonance imaging (fMRI) food picture evaluation task in non-obese men (Byrne et al., 2016). In parallel, the subjective appeal of high-energy food pictures decreased and energy intake during an *ad libitum* meal was reduced. These results support the mediating role of SCFAs in microbiota-gut-brain axis communication. Moreover, our group recently showed that daily administration of known amounts of a SCFA mixture equivalent to 20 or 10 g of AX (174.2 mmol acetate, 13.3 mmol propionate, and 52.4 mmol butyrate, or half the dose, respectively) to the colon of healthy men for 1 week significantly reduced the cortisol response to an acute psychosocial stress challenge (Dalile et al., 2020). Furthermore, we found that the increase in circulating SCFAs was associated with a decrease in cortisol response to stress. This preliminary study indicates that SCFAs are clearly an important mechanism through which fermentable fibres can affect HPA axis (a major neuroendocrine system that regulates stress responses as well as affective processes) reactivity to stress.

Conceptually, SCFAs may influence affective and cognitive processes by influencing multiple pathways that have been reviewed elsewhere (Dalile et al., 2019). However, the extent to which increasing the substrate of SCFAs (soluble fibres) increases SCFA concentrations and consequently influences affective and cognitive processes, remains understudied. In the following sections, we focus on potential mechanisms by which fibre consumption may alter affective and cognitive processes via interactions with SCFAs and other microbiota-gut-brain axis mediators, namely, the immune system, the intestinal barrier, and hypertension. As studies that directly investigate the effect of dietary fibre on SCFAs and these mediators and subsequent changes in affective and cognitive processes are currently lacking, we summarise the available evidence for an effect of SCFAs on these intermediate systems and for the involvement of these systems in the regulation of affective and cognitive processes.

#### Immune system (indirect effects)

##### Immune system and cognitive and affective processes

The immune system is important for both affective and cognitive processes. Challenging the immune system activates cytokines which are involved in the repair of damaged tissue and the restoration of homeostasis. The inflammatory response may also elicit deleterious effects, such as alterations in mood and cognition. This became evident from, for instance, a study vaccinating healthy volunteers with *Salmonella typhi* which increased IL-6, IL-1Ra, and tumour necrosis factor (TNF)-α, but also decreased mood without any signs of physical sickness (Wright et al., 2005). Other studies using acute inflammatory stimulation showed similar results (Brydon et al., 2009; Strike et al., 2004). Also, endotoxin-induced inflammation showed a dose-dependent elevation in pro-inflammatory cytokines IL-6 and TNF-α, but also anti-inflammatory cytokine IL-10 (Grigoleit et al., 2011). These elevations were accompanied by a dose-dependent increase in anxiety, negative mood, and poor long-term memory. Involvement of inflammation in negative mood and poor cognitive function is supported by numerous reviews that repeatedly showed an increased incidence of negative mood symptoms with elevated levels of pro-inflammatory markers (CRP, TNF-α, IL-1β, IL-2, and IL6) in peripheral blood and cerebrospinal fluid (CSF) in patients with MDD (Dowlati et al., 2010; Hannestad et al., 2011; Hiles et al., 2012). Regarding cognition, higher levels of CRP are associated with impairment in several cognitive domains such as with lower psychomotor speed and poorer executive function (Krogh et al., 2014).

Reducing inflammation has been shown to improve mood (Allison and Ditor, 2015). Moreover, mood stabilisers such as lithium and valproate suppressed IL-6 levels in patients with bipolar disorder compared to untreated patients (Kim et al., 2007). Atypical antipsychotics, which are used as a treatment for both MDD and bipolar disorder, have also been shown to decrease levels of TNF-α and IL-6 in animal models (Bian et al., 2008; Kato et al., 2007). Furthermore, meta-analyses assessing various cytokine levels after anti-depressant treatment in people with depression show significant decreases in IL-6, IL-1β, and CRP (Hannestad et al., 2011; Hiles et al., 2012). It seems therefore likely that inflammation plays a significant role in mood. To this end, reducing inflammation may aid negative mood symptoms in some individuals.

##### SCFAs and immune system

SCFAs are able to modulate the immune system. SCFAs may reduce inflammation by modulating molecular signalling pathways, including free fatty acid receptor (FFAR) 2 and 3 activation (Vinolo et al., 2011), and histone deacetylation (HDAC) inhibition (Waldecker et al., 2008). As SCFAs directly interact with FFAR2 on immune cells, which is suggested to regulate regulatory T-cells (T_reg_ cells) in the colon (Smith et al., 2013), T_reg_ cells may then suppress immune responses, thereby reducing inflammation.

Of all SCFAs, butyrate seems the most potent SCFA in modulating the immune system. Butyrate increases the expression of anti-inflammatory molecules in dendritic cells (DCs) and macrophages, thereby supporting T_reg_ differentiation (Singh et al., 2014), and hence suppress inflammation. Furthermore, due to its ability to inhibit HDAC activity, butyrate decreases the secretion of proinflammatory cytokines interleukin (IL)-12 and IL-6 in dendritic cells and allows dendritic cells to promote anti-inflammatory cytokine IL-10-secreting T cells (Chang et al., 2014). Moreover, butyrate inhibits the production of pro-inflammatory cytokines interferon (IFN)-y and IL-2 (Looijer-Van Langen and Dieleman, 2009). Propionate may also modulate inflammation by inducing forkhead box P3 (FOXP3) expression, a protein that regulates the development and function of T_regs_ (Arpaia et al., 2013). Furthermore, both propionate and acetate have been found to increase the production of anti-inflammatory cytokine IL-10 (Cavaglieri et al., 2003).

Indeed, most animal studies using prebiotics indicate beneficial effects on the immune system by lowering proinflammatory cytokine expression. For instance, gene expression of pro-inflammatory cytokines IL-1β and (TNF)-α decreased in the brain of mice fed with a high fibre (5 per cent inulin) diet but not in mice fed a low fibre (1 per cent cellulose) diet (Matt et al., 2018). The decrease in IL-β and TNF-α correlated significantly with faecal butyrate levels, with higher butyrate levels corresponding with lower pro-inflammatory gene expression. Furthermore, 3-week administration of a FOS + GOS mixture (dissolved in drinking water for 0.3–0.4 g/mouse/day) reduced the elevations in proinflammatory cytokine levels (IL-6 and TNF-α) caused by chronic stress induction in C57BL/6J male mice (Burokas et al., 2017). Moreover, mice fed with a high fermentable fibre (10 per cent pectin) diet became less sick and recovered faster from lipopolysaccharide (LPS)-induced sickness compared to mice fed with non-fermentable fibre (5 per cent cellulose) (Sherry et al., 2010). LPS-stimulated macrophages from the mice fed with pectin showed decreased pro-inflammatory cytokines IL-1β, TNF-α, interferon (IFN)-γ, IL-12, and nitrate, and increased anti-inflammatory cytokine IL-1RA compared to mice fed with cellulose. With regards to brain-based inflammation levels, increased levels of anti-inflammatory IL-1RA and IL-4 mRNA, and a decrease in pro-inflammatory IL-1β and TNF-α were observed in mice fed with pectin compared to mice fed with cellulose (Sherry et al., 2010). Since IL-4 expression is stimulated by histone acetylation, the authors hypothesised that the increased butyrate concentrations observed as a result from dietary fibre fermentation of pectin stimulated IL-4, and hence may aid immune response regulation (McLoughlin et al., 2017).

In humans, few studies have administered SCFAs and found an effect on inflammation as shown by a systematic review where only two of five studies revealed statistically significant decreases in serum inflammatory markers, namely, decreases in IL-1β following colonic infusion of SCFA mixture, and decreases in TNF levels following rectal acetate administration. Some studies using prebiotics support the hypothesis that SCFAs induce immunomodulatory effects SCFAs. For instance, in healthy elderly, consumption of a mixture of GOS (B-GOS; 5.5 g/day) for 10 weeks increased levels of anti-inflammatory cytokine IL-10, and lowered levels of pro-inflammatory cytokines IL-1, IL-6, and TNF-α (Vulevic et al., 2008). Furthermore, supplementation with inulin and xylooligosaccharides (XOS; 6.64 g/day) for 4 weeks lowered the expression of pro-inflammatory IL-1b, IL-8, IL-12, and TNF-α, whereas it increased anti-inflammatory IL-10 and IL-13 in the blood of healthy subjects (Lecerf et al., 2012). In a recent meta-analysis, 9 out of 13 studies reported a significant decrease in one or more systemic pro-inflammatory cytokine (primarily TNF-α, IL-6, c-reactive protein (CRP), or IFN-γ) after consumption of prebiotic oligosaccharides compared with control (McLoughlin et al., 2017). In contrast, in two studies that were conducted in healthy participants, CRP, TNF-α, and IL-6 increased following oligosaccharide supplementation. Another systematic review, conducted in 10 prebiotic and synbiotic (supplements combining probiotics and prebiotics) trials, of which 7 prebiotic and 3 synbiotic, representing 534 obese/overweight subjects, also found inconsistent effects of prebiotic treatment on immunomodulation. They found that only 6 out of 10 trials (two with GOS, one with inulin, and three with different synbiotics) reduced CRP levels, four out of four trials (one with oligofructose-enriched inulin, one with inulin, and two with different synbiotics) reduced levels of IL-6 and/or TNF, and four out of the five trials (one with oligofructose-enriched inulin, one with inulin, one with GOS, and one with different synbiotics) lowered endotoxin levels (Fernandes et al., 2017). Taken together, it might still be too early to promote the use of prebiotics for reduction of low-grade inflammation. However, the evidence seems promising and more studies are needed to conclude whether prebiotics can lower inflammation via increased SCFA production in humans, and in turn promote beneficial effects on mood and cognition.

#### Intestinal barrier

##### Intestinal barrier and cognitive and affective processes

The intestinal barrier is mainly responsible for regulating the absorption of nutrients, electrolytes, and water from the lumen, and to prevent pathogenic microorganisms and toxic luminal substances to enter the host. A dysfunctional intestinal barrier may lead to increased intestinal permeability, and hence to the translocation of luminal antigens, bacteria, and toxins through the intestinal wall into the blood stream. In turn, this may cause low-grade inflammation (Caspani and Swann, 2019) and hence may affect affective and cognitive processes. Indeed, some studies report associations between depressive symptoms and increased levels of indirect markers of intestinal permeability such as intestinal-type fatty acid-binding protein (I-FABP), fatty acid-binding protein 2 (FABP2), LPS, and zonulin (Ohlsson et al., 2019; Stevens et al., 2018). Moreover, a study using a direct measure of intestinal permeability (lactulose to mannitol ratio) also found a positive association with depression severity (Calarge et al., 2019).

##### SCFAs and intestinal barrier

SCFAs are the preferred energy substrates for the colonic epithelium (Cushing et al., 2015) and can contribute to enhanced intestinal barrier function. This has been shown in *in vitro* and in *in vivo* experiments in which the application of individual and combinations of SCFAs increased transepithelial electrical resistance (Feng et al., 2018; Suzuki et al., 2008), decreased paracellular transport markers (Suzuki et al., 2008), and stimulated the formation of tight-junctions (Feng et al., 2018). Furthermore, SCFAs may protect the intestinal barrier from disruption induced by LPS through the inhibition of the NLPR3 inflammasome and autophagy (Feng et al., 2018).

Interestingly, an experimental study showed that 1-week oral administration of a SCFA mixture (67.5 mM acetate, 25 mM propionate, and 40 mM butyrate) alleviated stress-induced increases in intestinal permeability and decreased depressive-like behaviour in mice (Van De Wouw et al., 2018). Moreover, animal studies show that inulin supplementation (0.5 per cent as well as 1 per cent) increased expression of intestinal barrier function-related genes after *Salmonella enterica* infection in SPF chickens (Song et al., 2020). In obese mice, oligofructose increased the relative abundance of *Bifidobacterium* spp., leading to increases in GLP-2 and tight-junctions, lower levels of inflammation, and a significant improvement of intestinal permeability (Cani et al., 2009). As ITFs stimulate SCFA production, the effect is likely mediated by SCFAs, although this is speculative as SCFA concentrations were not measured.

Human studies that evaluated the impact of dietary fibre on intestinal permeability yielded inconsistent results. Administration of green bananas and pectin for 1 week significantly reduced intestinal permeability and stool output in children with diarrhoea (Rabbani et al., 2001). Another study in healthy subjects found a significant reduction in intestinal permeability markers zonulin and GLP-2 after consumption of inulin-pasta (8 weeks) compared to control pasta (Russo et al., 2012). In contrast, a high dose of inulin (30–35 g/day) for 1 week in patients on liquid enteral nutrition did not affect intestinal permeability (Sobotka et al., 1997) neither did supplementation with GOS (5.5 g/day) for 12 weeks in men with well-controlled type 2 diabetes (Pedersen et al., 2016).

These results suggest that the beneficial effects of SCFA on intestinal permeability are not always evident from human studies. Fermentable fibres may induce beneficial effects on affective and cognitive processes via stimulating SCFA production. However, there is a need for human studies that assess whether improvements in intestinal barrier function by means of SCFA stimulation are also associated with improvements in mood and cognition, albeit by attenuating stress-induced negative effects, to confirm this speculation.

#### Hypertension

##### Hypertension and cognitive and affective processes

Hypertension, also known as high blood pressure, is a well-established risk factor for cognitive impairment and dementia (Forte et al., 2020). Cognitive impairment is more frequent in patients with hypertension, and this is positively associated with the severity (stage) of hypertension (Muela et al., 2017). Moreover, the prevalence of anxiety disorders is higher in patients with essential hypertension (Vetere et al., 2007). However, it remains to be established to what extent a reduction in blood pressure improves affective processes and cognition. A systematic review and meta-analysis of randomised controlled trials (RCTs) indicated that pharmacological treatment of hypertension reduces cognitive decline in older adults (Gupta et al., 2020). On the other hand, recent large-scale associative studies report mixed findings. In 14,195 hypertensive older adults from the United States and Australia, the use of beta blockers alone or combined with angiotensin-receptor blockers was associated with depressive symptoms, but this was not the case when using angiotensin-receptor blockers on its own, angiotensin-converting enzyme inhibitors, calcium channel blockers, or any other possible combination of drugs among them (Agustini et al., 2020). However, another cross-sectional study comprising 1.8 million patients from Scotland reported that angiotensin antagonists and calcium channel blockers were associated with episodes of MDD in hypertensive patients with or without history of mood disorders (Shaw et al., 2020). Another nation-wide population-based study of 5.4 million people in Denmark found that no drugs belonging to angiotensin agents, calcium antagonists, β-blockers, or diuretics were associated with an increased risk of depression. In contrast, angiotensin agents, calcium antagonists, and β-blockers were associated with decreased rates of depression. Moreover, non-clinical evidence indicates that mood and blood pressure are related, with increasing intensity of negative moods (stress, anxiety, and anger) being associated with increased blood pressure, with feeling tired (low energy) being associated with the opposite pattern, while changes in intensity in positive mood (happy) showing little changes in blood pressure (Shapiro et al., 2001).

##### SCFAs and hypertension

Meta-analyses and reviews of RCTs reveal that higher dietary fibre consumption reduces blood pressure, an effect that is most pronounced in patients with hypertension, regardless of the type of fibre (Aleixandre and Miguel, 2016). Some studies report differences in gut microbiota composition, particularly in SCFA-producers, as well as in SCFA profiles in spontaneously hypertensive rats (SHR) and in hypertensive patients (Yang et al., 2015). For example, patients with high blood pressure exhibit significantly less butyrate-producers such as *Roseburia* and *Eubacterium* but also lower plasma butyrate concentrations compared to normotensive subjects (Kim et al., 2018). Furthermore, intraperitoneal or intramedullary butyrate administration (Kim et al., 2018; Wang et al., 2017), acetate (Marcques et al., 2017), and propionate (Bartolomaeus et al., 2019) administration in drinking water resulted in anti-hypertensive effects in rodents. Most recently, expression levels of butyrate‐sensing receptors FFAR3 and olfactory receptor (OLFR) 59 in the hypothalamus of SHR were found to be lower than in control rats, likely underlying the reduced effects of centrally administered butyrate on blood pressure in the SHR. Finally, functional magnetic resonance imaging revealed reduced activation of cardio regulatory brain regions of SHR compared to controls following ICV injection of butyrate to reach physiological concentrations (3–5 μM/L) in the CSF (Yang et al., 2019). Only correlational evidence exists in humans, which indicate that higher SCFA-producing bacteria are associated with lower blood pressure and high faecal SCFA levels (De La Cuesta-Zuluaga et al., 2019; Verhaar et al., 2020). Studies that aim to reduce hypertension in humans by supplementing prebiotics or other dietary fibres would benefit from quantifying SCFAs to determine the extent to which they mediate the effect of these dietary interventions on blood pressure. These studies suggest that more research is needed to understand the role of hypertension in mood and cognition, and non-pharmacological interventions such as dietary fibre or SCFA supplementation may be ripe targets for such research.

#### Brain-derived neurotrophic factor

BDNF is a nerve growth factor found in the brain and in the periphery with an important function in normal neural development and in long-term memory. It is considered as a potential marker for neuronal integrity and brain functions. As central and peripheral BDNF are highly correlated in rats (*r* = 0.86) (Harris et al., 2016), circulating levels of BDNF may be used as an appropriate measure for brain levels of BDNF. Dietary fibre may indirectly increase circulating BDNF levels via modification of the microbiota, in particular levels of *Bifidobacterium* and *Lactobacillus.* Increases in the relative abundance of these strains and consequent increases in BDNF levels were accompanied by a reduction in depressive symptoms as well as improvements in cognitive performance in both animal (Bercik et al., 2010, 2011) and human studies (Haghighat et al., 2019).

Dietary fibres have indeed shown to increase BDNF levels in both animals and humans, and some already found associations with improvements in cognition. For instance, in a rat study administering placebo, inulin, *E. faecium*, or *E. faecium +* inulin, BDNF levels only increased in the probiotic and synbiotic group, but not in the prebiotic group, with only the synbiotic group showing improved memory (Romo-Araiza et al., 2018). Furthermore, in healthy subjects, an evening meal consisting of rye kernel bread increased plasma BDNF levels by 27 per cent at fasting on the next morning compared to white wheat bread (Sandberg et al., 2018). Consumption of the same bread for three consecutive days was associated with a higher abundance of *Prevotella* compared to the white wheat bread (Prykhodko et al., 2018), and the *Prevotella* genus was positively associated with plasma levels of BDNF. Unfortunately, no plasma SCFAs were measured which prevented evaluating the relationship between SCFAs and BDNF. Despite evidence that butyrate increases acetylation around the promoters of BDNF, thereby increasing its transcription (Intlekofer et al., 2013) and the myriad pre-clinical studies showing that it increases following butyrate administration (Stilling et al., 2016), our recent study in healthy subjects showed that colonic administration of SCFAs did not alter serum BDNF levels (Dalile et al., 2020). Together, the evidence thus far seems to indicate that the relationship between dietary fibre consumption and BDNF levels is mediated by changes in gut microbiota composition, rather than increases in colonic or circulating SCFAs.

### Direct effects of dietary fibre on the immune system

#### Dietary fibre and immune system

Few studies explored immune effects of dietary fibres that are independent of the gut microbiota. A recent study demonstrated that dietary cellulose supplementation modulates the immune response. Mice were fed either a normal diet or a high-fibre cellulose diet for 2 weeks and were, subsequently, injected with endotoxin (LPS). Mice fed the high-fibre cellulose diet showed lower levels of proinflammatory cytokines (IL-1α, IL-1β, and IFN-α), lower numbers and activation of splenic macrophages and DCs, and hyporesponsiveness of T cells (Di Caro et al., 2019). Splenic macrophages and DCs are innate effector cells that are crucial for the host defence to protect against bacterial infections (Di Caro et al., 2019). Since cellulose is hardly fermentable, the observed effects likely arose from a direct interaction of the fibre with the immune system, rather than from SCFA production.

Also, other glucans, more specifically β-1,3/1,6-glucans, modulate the innate and acquired immune system. Those compounds bind to pattern recognition receptors including complement receptor 3, scavenger receptors, lactosylceramide, and dectin-1, which are expressed on cells of myeloid origin, including macrophages, dendritic cells, and neutrophils (Murphy et al., 2010). Although small amounts of β-glucan may be absorbed after oral administration, it is most likely that β-glucan primarily act in the gut epithelium. β-Glucan has been shown useful in the prevention of treatment of allergic disease. No studies to date have evaluated whether the impact of β-glucan on the immune system results in improvements in cognitive or affective processes.

### Cholesterol

#### Cholesterol and cognitive and affective processes

Cholesterol is important for normal brain function and our mental well-being as well as cognition. However, its involvement is complex. Cholesterol demand by neurons in the brain is very high, since it is implicated in a variety of neuronal processes such as neurite formation and synaptic activity. Several studies found that higher levels of LDL cholesterol were associated with depressive mood in men (Kim et al., 2019; Tedders et al., 2011) whereas low levels of high-density lipoprotein (HDL) cholesterol were significantly associated with depression symptoms in women (Tedders et al., 2011) and men (Lehto et al., 2010), and is a potential risk factor for developing a mood disorder in females (Kim et al., 2018). Up to 44 per cent of the subjects who were diagnosed with a mood disorder suffered from mild hypercholesterolemia (>5.2 and 6.2 mmol/L) and 21 per cent from hypercholesterolemia (>6.2 mmol/L) (Davison and Kaplan, 2012). Importantly, dietary cholesterol cannot cross the blood brain barrier. Therefore, the mechanisms by which cholesterol influences learning and memory are thought to combine peripheral (atherogenic and proinflammatory) and central (accumulation of intracellular beta amyloid) effects.

There is some evidence that pharmacological reduction of plasma cholesterol with statins, the most common cholesterol-lowering drugs, may improve cognitive function in subjects without dementia (Schreurs, 2010). However, the effects of statins on mood modulation remain controversial. For instance, chronic statin treatment in rats (10 mg/kg atorvastatin; 10 mg/kg simvastatin; 30 mg/kg pravastatin) reduced anxious behaviour in the open-field task (Citraro et al., 2014; Wang and Gao, 2009). Preclinical studies using the forced swimming test also support the anti-depressant effects of statin treatment (Can et al., 2012). Another study demonstrated that statin treatment decreased the risk of depression (Yang et al., 2003). In contrast, low dose statin treatment (simvastatin 1 mg/kg; atorvastatin 0.5 mg/kg) did not improve spatial memory and learning in guinea pigs, but instead increased levels of anxiety (Maggo et al., 2012). Furthermore, some studies in humans found that depressive symptoms increased rather than attenuated after statin treatment (Lechleitner et al., 1992; Morales et al., 2006). It is possible that lowering cholesterol induces positive effects on cognitive and affective processes by lowering blood pressure (see section “Hypertension”).

#### Dietary fibre and cholesterol

Soluble rather than insoluble fibres possess cholesterol lowering abilities in humans. The mechanism is most likely related to the viscous properties of soluble fibre. By increasing the viscosity in the small intestine, reabsorption of bile acids is reduced, in turn leading to increased *de novo* synthesis of bile acids from cholesterol in the liver and lower circulating cholesterol levels (Fuller et al., 2016). The role of β-glucan in lowering cholesterol has been extensively documented and led the U.S. Food and Drug Administration (FDA) to authorise the use of health claims on oat-containing products, stating that consumption of β-glucans at least 3 g per day lowers the risk of cardiovascular disease (Food and Drug Administration, 2019). These health claims have also been approved by the European Commission, as well as in various other jurisdictions (European Commision, 2011, 2012). A meta-analysis showed that also pectin, soluble fibre from psyllium, and guar gum have cholesterol-lowering abilities (Brown et al., 1999). Viscous fibres should be utilised in intervention studies to test their effects of affective and cognitive processes, and if supported, investigate whether their cholesterol-lowering ability drives the effects on brain function in humans.

## Conclusion

Collectively, studies suggest that dietary fibres may be promising in inducing psychological changes in cognitive and affective processes. The evidence base indicates that fermentable fibres, such as ITFs, β-glucans, pectin, and gums, are the strongest candidates to modulate these psychological functions. This is mainly due to their ability to modulate the microbiota and increase SCFA production. However, their ability to increase BDNF levels may also reinforce their effects on cognitive and affective processes. On the other hand, viscous fibres such as β-glucan and non-fermentable fibres such as cellulose may exert beneficial effects on cognitive and affective processes via microbiota-independent mechanisms such as lowering cholesterol and inflammation, respectively. Notably, a research gap remains, where the effects of different types of fibres on mood and cognition via the proposed mechanisms still needs to be investigated. In addition, while preclinical studies of dietary fibres on gastrointestinal mechanisms are ample, well-designed studies of dietary fibre effects on mood and cognition with simultaneous assessment of putative mechanisms of actions in humans are needed. The aim with this review was to outline potential additional microbiota-dependent and independent mechanism through which different fibre properties can act to affect affective and cognitive processes in order to give a more complete picture of the effects of dietary fibres and to help explain additional variance in inter-individual psychological responses to fibre consumption. The resolution of various mechanistic and functional links outlined here may better facilitate personalised nutrition for maintaining optimal psychological functioning in states of health or disease.

## Data Availability

Data sharing is not applicable to this article as no new data were created or analysed.
